# FM807, a curcumin analogue, shows potent antitumor effects in nasopharyngeal carcinoma cells by heat shock protein 90 inhibition

**DOI:** 10.18632/oncotarget.14970

**Published:** 2017-02-01

**Authors:** Min Ye, Wei Huang, Wen-wei Wu, Yang Liu, Sheng-nan Ye, Jian-hua Xu

**Affiliations:** ^1^ School of Pharmacy, Fujian Medical University, Fuzhou 350004, China; ^2^ The First Affiliated Hospital of Fujian Medical University, Fuzhou 350004, China; ^3^ Fuijan Provincial Key Laboratory of Natural Medicine Pharmacology, Fuzhou 350004, China

**Keywords:** FM807, nasopharyngeal carcinoma, epidermal growth factor receptor, β-catenin, Hsp90 inhibitor

## Abstract

Nasopharyngeal carcinoma (NPC) is an epithelial malignancy usually associated with overexpression of both epidermal growth factor receptor (EGFR) and β-catenin. FM807 is a novel curcumin analogue with antitumor activity against both poorly and well-differentiated NPC cell lines as well as good selectivity for tumor cells. FM807 actions were shown to include inhibition of cell growth, induction of necrotic/late apoptotic cell death, and G1 arrest in NPC cells. Crucially, it exhibited potent antitumor effects both *in vitro* and *in vivo*. Binding of FM807 to the N-terminus of Hsp90 disrupted Hsp90/client complexes, resulting in degradation of the Hsp90 client protein EGFR and inhibition of the downstream Raf/MEK/ERK and PI3K/AKT pathway. FM807 also depleted levels of the intranuclear transcription factors β-catenin, Cyclin D1 and c-Myc levels by inhibiting Hsp90 chaperoned nuclear transport. In conjunction with its low toxicity in NPC xenograft mice, these results provide a sound preclinical basis for further development of FM807 as a novel therapeutic agent in the treatment of NPC.

## INTRODUCTION

Nasopharyngeal carcinoma (NPC) is a malignant tumor arising from the epithelia cells of the nasopharynx. High rates of NPC occur in Southeast Asia, especially southern China, with significant racial and regional distribution characteristics [1]. As early symptoms are not obvious, 60–70% of patients with NPC are not diagnosed until the disease reaches an advanced stage [2]. Radiotherapy and chemotherapy are the standard methods for NPC, and their clinical outcome have been greatly improved since the applications of intensity modulated radiotherapy (IMRT) [3]. Although several trials reported very encouraging results with locoregional control rate over 90%, clinicians are still puzzled by the local recurrence and/or distant metastasis as the major pattern of disease failure [4].

Heat shock protein 90 (Hsp90) is a highly conserved molecular chaperon involved in the maturation and stabilization of over 200 oncogenic client proteins [5]. Most Hsp90 client proteins, such as epidermal growth factor receptor (EGFR), AKT, C-Raf (also called Raf-1), Cdk4, Bcr-Abl, and p53, are essential for tumor growth, proliferation and survival [5–7]. The Hsp90 inhibitor AT13387 could inhibit NPC C666-1 cell growth and induced cellular senescence with the downregulation of multiple Hsp90 client oncoproteins EGFR, AKT, CDK4, and significantly suppressed tumor formation in C666-1 NPC xenografts [8]. Hsp90 inhibitor AUY922 combined with erlotimib could also overcome the EGFR mutant with acquired resistance to tyrosine kinase inhibitors (TKIs) [9]. The classical Hsp90 inhibitor 17-AAG could also downregulated mutant EGFR expression and sensitized EGFR mutant tumors [10]. Therefore, inhibition of the Hsp90 machinery is considered to be a potent strategy in NPC therapies.

Curcumin (Cur), a polyphenol derived from the herb *Curcuma longa*, has been extensively studied as chemopreventive agent ascribed to the modulation of signal-transduction pathways associated with cell proliferation, invasion and angiogenesis [11]. Cur has been reported to down-regulate some clients of Hsp90, such as BCR-ABL, AKT, EGFR, and dissociate the Hsp90 co-chaperone p23, suggesting an inhibition of Hsp90 function [12, 13]. Despite its poor bioavailability *in vivo*, Cur remains a good lead compound [14], as hundreds of curcumin derivatives have been reported to show lead-like properties and proving to be more active recently [15–18]. Therefore, a series of curcumin derivatives have been synthesized in our lab, and tested for their anti-tumor activies by dozens of cancer cells [19, 20]. We incorporated a 2-hydroxy-benzoic acid chain into Cur and synthesized a novel Cur analogue FM807 (2-hydroxy-, 4-[(1E, 6E)- 7-(4-hydroxy -3-methoxyphenyl)- 3,5-dioxo -1,6-heptadien-1-yl]- 2-methoxyphenyl ester), which retains the β-diketone structure and exhibits better anti-infalmmatory activity than that of Cur [21]. In the present work, we showed that FM807 inhibited the proliferation and induced G1 arrest in well-differentiated CNE1 and poorly-differentiated CNE2 cells *in vitro*, and exhibited antitumor activity in CNE2 xenograft *in vivo*. We demonstrated that FM807 physically bound to N-terminus of Hsp90 and inhibited Hsp90 function by affecting ATP-binding activity of Hsp90, leading to the degradation of Hsp90 client proteins including EGFR and downstream Raf/MEK/ERK and PI3K/AKT signal pathways. Moreover, subcellular fractionation of NPC cells provided information that differentially abundant intranuclear transcription factors such as β-cantenin would likely cause various chemosensitivity to CNE1 and CNE2, and the molecular chaperone function of Hsp90 for nuclear transport could also be inhibited by FM807. These data suggest that FM807 is a potent Hsp90 inhibitor against nasopharyngeal carcinoma cells.

## RESULTS

### FM807 inhibits proliferation of NPC cell lines

**Figure 1:**
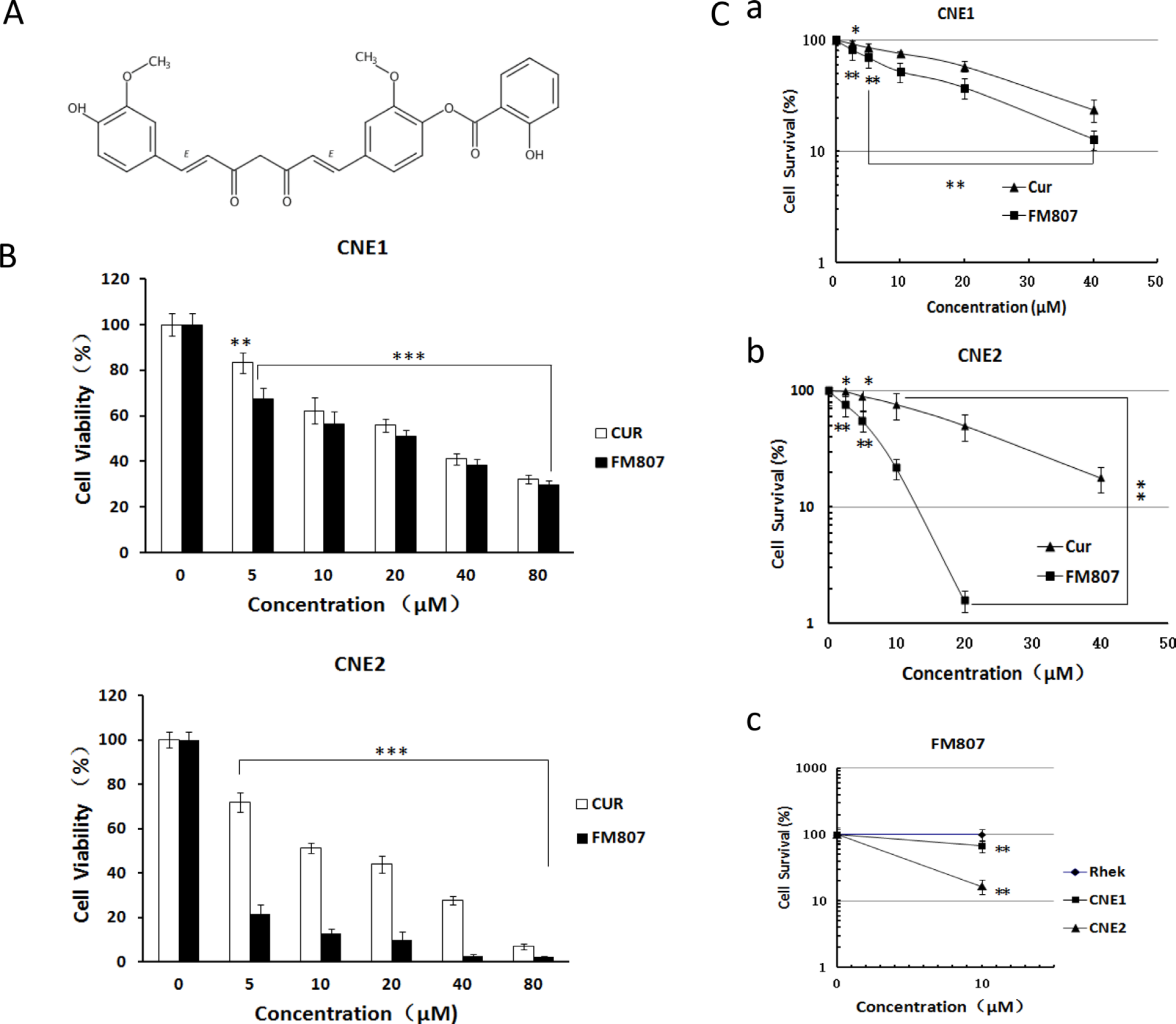
FM807 has a potent antiproliferation effect on NPC cell lines, but showed little impact on non-cancer Rhek cells (**A**) The chemical structure of FM807. (**B**) CNE1 and CNE2 cells were treated for 72 h in the presence of Curcumin and FM807 ranging from 0 to 80 μM. Cellular viability was measured using MTS assay and expressed as a percentage of vehicle-treated control. Results are presented as means ± SD of three independent experiments. *P < 0.05: significant difference from control by ANOVA; **P < 0.01, ***P < 0.001: very significant difference from control by ANOVA. (**C**) Effect of Curcumin and FM807 on the colony formation of (a) CNE1, (b) CNE2 and (c) Rhek cells. Quantification of the colony formation efficiency is presented as means ± SD of three independent experiments, *P < 0.05: significant difference from control by ANOVA; **P < 0.01, ***P < 0.001: very significant difference from control by ANOVA.

FM807 was synthesized as a derivative of Cur (Figure 1A). To evaluate its effectiveness on cellular proliferation, Cur and FM807 were administrated to the NPC cell lines CNE1 and CNE2 for 72 h, and subjected to an MTS assay (Figure 1B). The IC_50_ values of Cur and FM807 were determined to be 37.01 and 25.75 μM for CNE1, 15.33 and 3.59μM for CNE2, respectively. These indicated that the inhibitory action both Cur and its analogue FM807 on the poorly-dfferentiated NPC cell line CNE2 was stronger than on the well-differentiated CNE1, moreover FM807 was more potent than Cur. To confirm these findings, we next performed a colony formation assay to test effectiveness of Cur and FM807 on cell proliferation (Figure 1C 1a and 1b). These data also revealed that CNE2 cells were more sensitive to their effects. Importantly, FM807 displayed greater inhibition of cell proliferation than Cur in NPC cell lines.

To gain further insight into the efficacy of FM807, we next performed colony formation assays using the human normal epithelial cell line Rhek (Figure 1C, 1c). Although colony formation ability of non-malignant cells was lower than that of tumor cells, FM807 (10 μM) had no effect on Rhek colony formation (*P* > 0.05).

### FM807 induces CNE1 and CNE2 cell cycle arrest and apoptosis

**Figure 2:**
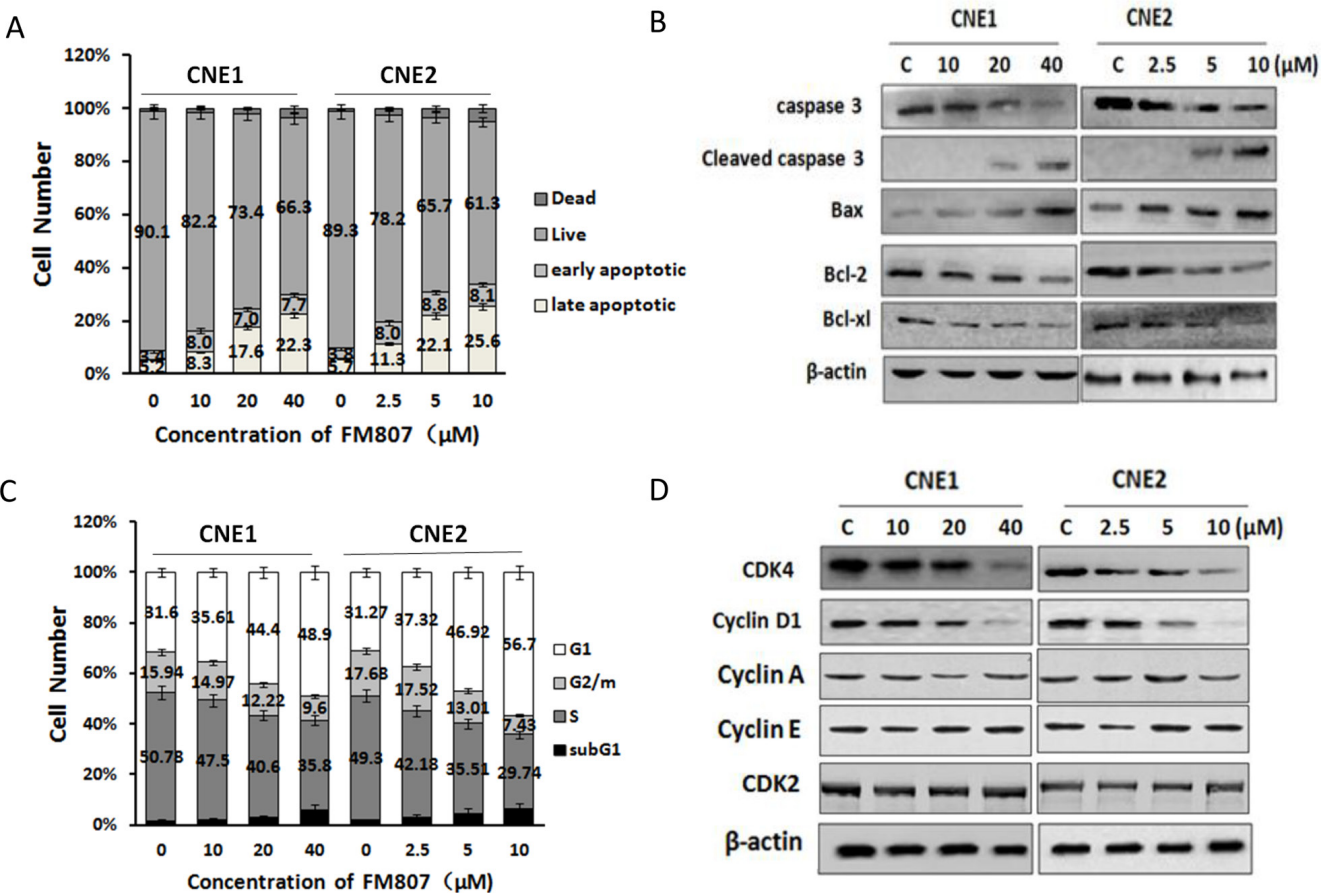
FM807 induces CNE1 and CNE2 cell cycle arrest and apoptosis (**A**) CNE1 and CNE2 cells were treated with FM807 for 24 h, and apoptotic cell death was detected by staining cells with an Annexin-V: FITC Apoptosis Detection Kit for later flow cytometry analysis. (**B**) CNE1 and CNE2 cells were treated with increasing doses of FM807 for 24 h, and apoptosis signal proteins were detected using western blot analysis, β-actin was used as a loading control. (**C**) CNE1 and CNE2 cells were treated with increasing doses of FM807 for 24 h. Cells were then fixed with 70% ethanol at –20°C overnight, incubated with RNase A at 37°C for 30 min, stained with propidium iodide for 10 min, and analyzed with flow cytometry. (**D**) CNE1 and CNE2 cells were treated with increasing doses of FM807 for 24 h, and cell cycle signal proteins were detected using western blot analysis.

We next assessed the effectiveness of FM807 on inducting cell death in both CNE1 and CNE2 cells. Cells from both lines were treated with FM807 for 24 h and analyzed for apoptotic cell death using the FITC: Annexin-V Apoptosis Detection Kit I. Results showed a dose-dependent induction of apoptosis that had an obvious necrotic/late apoptotic cell death in FM807-treated cells (Figure 2A, *P* < 0.05). Western blot analysis showed that anti-apoptotic proteins (Bcl-2 and Bcl-xl) exhibited similar reductions, which were accompanied by increases in the levels of pro-apoptotic proteins (Bax and cleaved caspase-3) (Figure 2B), suggesting that the induction of apoptosis is one of the major anti-tumor mechanisms of FM807 in CNE1 and CNE2 cells.

When compared to vehicle-treated controls, FM807-treated cells displayed showed marked G1 phase arrest after 24 h of treatment. The increase in the G1 phase cell population induced by FM807 was accompanied by a concomitant decrease in cells in S and G2/M phases. Moreover, FM807-treated cells were more remarkable at inducing G1 arrest (Figure 2C, *P* < 0.05). We next turned to western blot analysis to further investigate the molecular mechanisms involved in FM807-induced cell cycle arrest of CNE1 and CNE2 cells. CDK2, Cyclin A and Cyclin E showed no significant change with FM807 exposure. Since the induction of the cell cycle regulator CDK4 and its cognate cyclin, Cyclin D1, by mitogenic signals is a critical event in the G1/S transition [25], the observed G1 arrest was consistent with down-regulation of Cyclin D1 and CDK4 (Figure 2D).

### FM807 blocks EGFR-mediated downstream signaling pathways

Aberrant EGFR overexpression is frequently observed in NPC, and is associated with tumor metastasis, recurrence, and poor survival in NPC patients [26–28]. Binding of the EGF ligand to the extracellular domain of EGFR activates the receptor and its downstream signaling pathways, ultimately activating or modulating various cellular processes [29]. Therefore, targeting EGFR has been suggested as a potent strategy for NPC therapy [30, 31].

**Figure 3:**
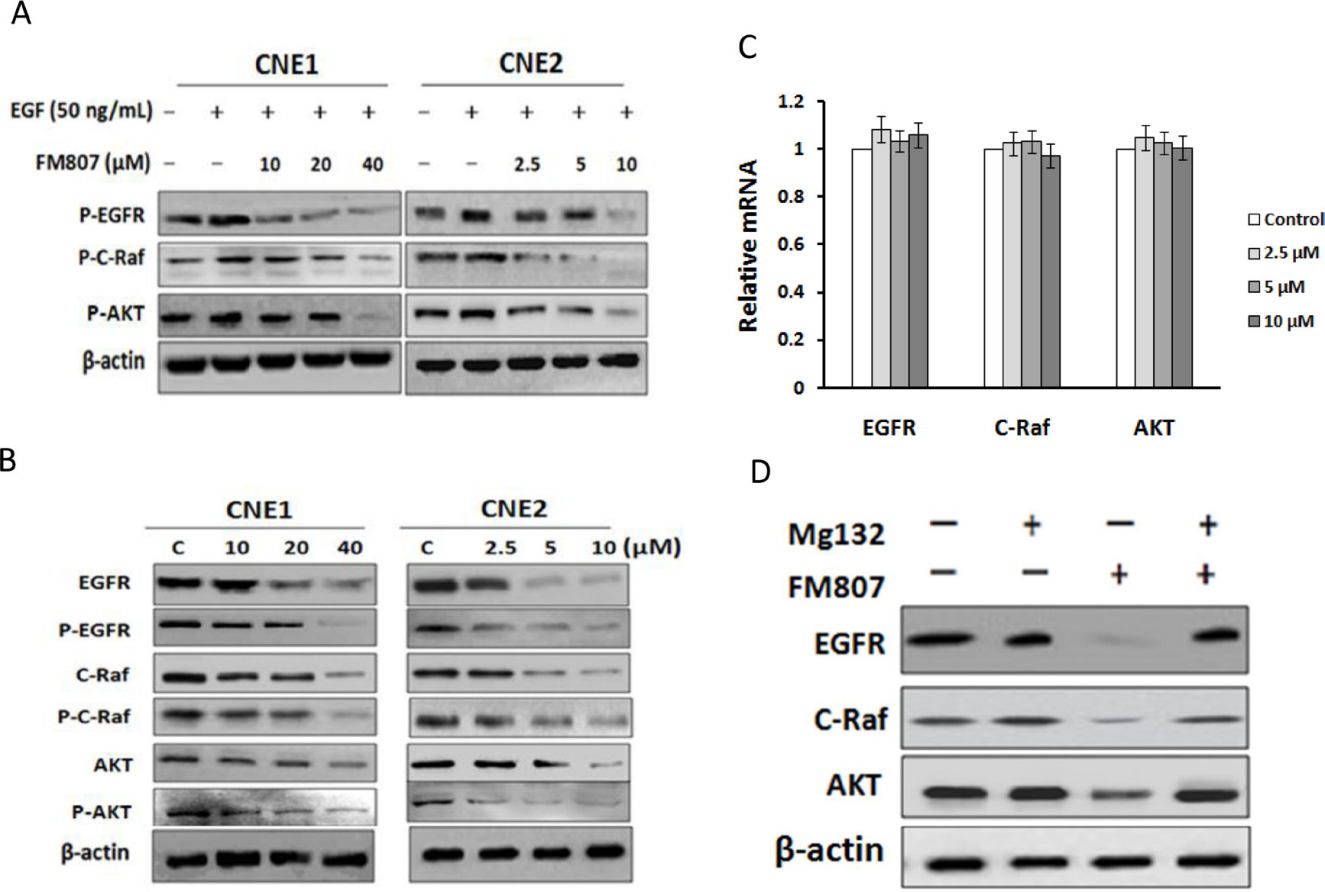
FM807 inhibits EGFR-mediated downstream signaling pathways (**A**) Serum-starved CNE1 and CNE2 cells were stimulated with 50 ng/mL EGF for 30 min, and then treated with FM807 for 24 h. The levels of the phosphorylation forms of EGFR (P-EGFR), C-Raf (P-C-Raf), and AKT (P-AKT) were analyzed using western blot. (**B**) Non-stimulated CNE2 cells were treated with FM807 for 24 h; EGFR, P-EGFR, C-Raf, P-C-Raf, AKT, and P-AKT protein levels were analyzed with western blot. (**C**) CNE2 cells were treated with FM807 for 24 h. Total RNA was extracted for quantitative real-time PCR of EGFR, C-Raf and AKT, using GAPDH as control. (**D**) CNE2 cells were pretreated with 1 μM of MG132 for 2 h in the presence or absence of 20 μM of FW807 for an additional 12 h. Whole-cell lysates were subjected to western blot analysis using antibodies against EGFR, C-Raf, AKT, and β-actin.

To gain a better understanding of the mechanisms behind FM807, we tested the effect of FM807 on activation of EGFR as well as the downstream Raf/MEK/ERK and PI3K/AKT pathways in EGF-stimulated and unstimulated CNE1 and CNE2 cells (Figure 3A and 3B). As expected, EGF activated EGFR as well as the Raf/MEK/ERK and PI3K/AKT pathways (see in lane 2). However, FM807 reduced levels of EGFR, C-Raf (also called Raf-1), and AKT in both unstimulated and EGF-stimulated CNE1 and CNE2 cells treated with increasing FM807 concentrations. Their phosphorylated forms also decreased in a similar manner by the growing dose of FM807. Quantitative real-time PCR analysis revealed that FM807 did not block transcription of EGFR, C-Raf or AKT (Figure 3C) in either cell line, indicating that the degradation was occurring at the level of protein-protein interaction.

Finally, the degradation was completely blocked with treatment of the proteasome inhibitor MG132, indicating that the proteasomal system was responsible for FM807-induced protein degradation (Figure 3D).

### Antitumor activity of FM807 in xenograft models *in vivo*

**Figure 4:**
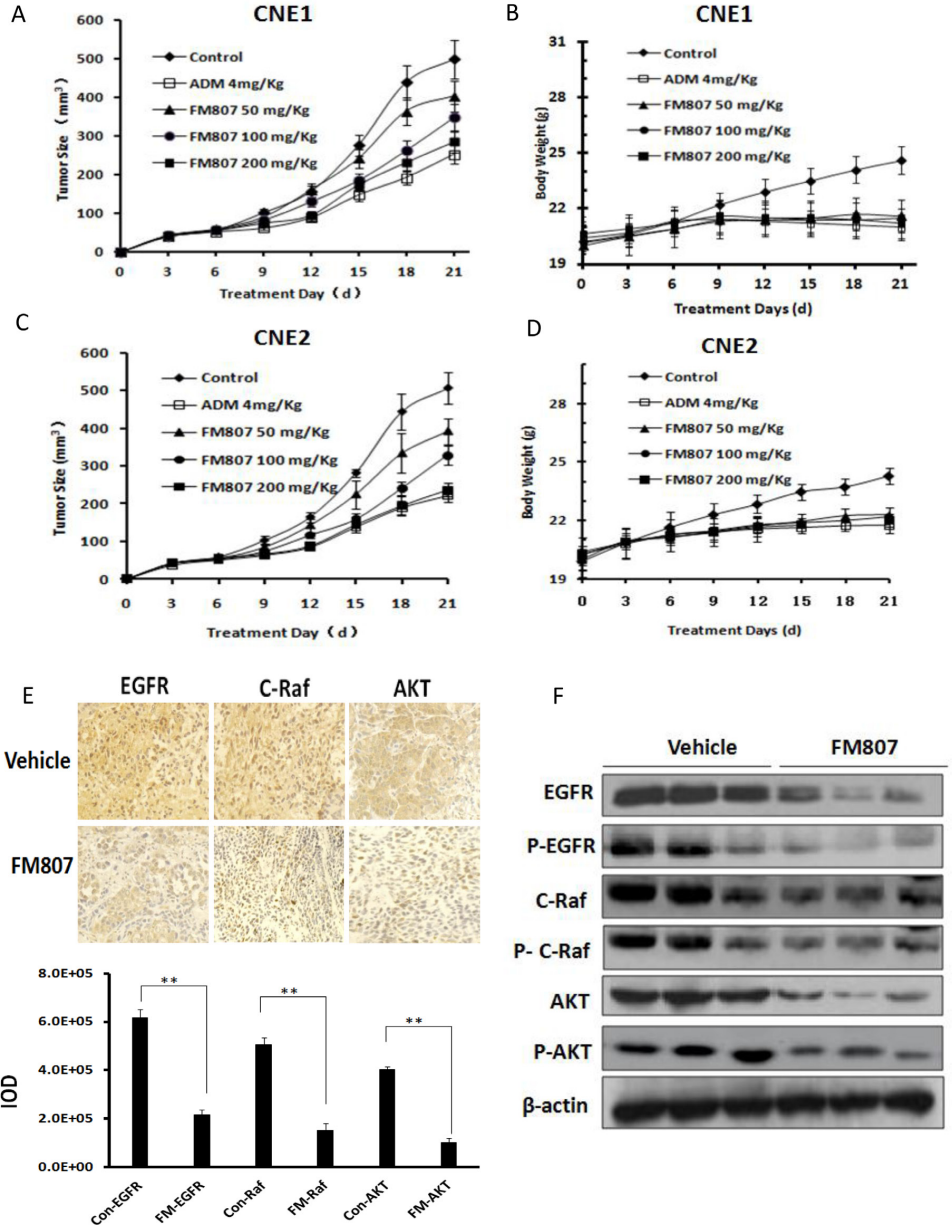
Antitumor activity of FM807 in xenograft models *in vivo* (**A**) CNE1 and (**C**) CNE2 tumor xenograft nude mice were randomized into vehicle or treatment groups (*n* = 6/group) and received FM807 at doses of 50, 100, 200 mg/kg i.g., (incorporated drug solid dispersions with poloxamer188, and then diluted with saline) for three weeks. Tumors volume was measured every three days to evaluate the anticancer activity of FM807. Data are presented as means ± SD (*n* = 6, *P* < 0.05). (**B**) CNE1 and (**D**) CNE2 tumor xenograft mouse body weight was measured every three days. Data are presented as means ± SD (*n* = 6, *P* < 0.05). (**E**) FM807 decreased the expression of EGFR, C-Raf and AKT in CNE2 tumors. “Vehicle” shows the tumor of mice treated by the vehicle; “FM807” represents the tumor of mice treated by 200 mg/kg FM807. (**F**) Tumor tissues excised from the CNE2 xenograft mice were lysed and changes in the levels of in EGFR, C-Raf, AKT and their phosphorylation forms were tested.

We next turned to CNE1 and CNE2 xenograft models to assess the antitumor effects of FM807 *in vivo*. Nude mice were injected subcutaneously with 1 × 10^7^ CNE1 or CNE2 cells. After injection, tumor-bearing mice were randomly assigned to one of three experimental groups (50, 100 and 200 mg/kg FM807, i.g.) or vehicle control group. FM807 inhibited tumor growth in CNE1 and CNE2 xenograft models in a dose-dependent manner (Figure 4A and 4C). When compared with the vehicle group, 50, 100 and 200 mg/kg FM807 treatment decreased tumor growth by 19.1% (*P* = 0.009), 30.3% (*P* = 0.007), and 42.9% (*P* = 0.006), respectively, in the CNE1 xenograft model (Figure 4A). Similarly, 50, 100 and 200 mg/kg FM807 treatment decreased tumor growth by 22.5% (*P* = 0.008), 35.1% (*P* = 0.005) and 53.3% (*P* = 0.006) in the CNE2 xenograft model (Figure 4C). All animals in the treatment groups survived without appreciable weight loss during the experiment (*P* > 0.05) (Figure 4B and 4D). Later histological analysis of lung, liver, heart, and kidney tissue obtained from treated mice showed no abnormalities after drug treatment (data not shown).

As shown by immunohistochemical and western blot analyses, FM807 induced a significant decrease in EGFR as well as inhibited downstream Raf/MEK/ERK and PI3K/AKT pathways in CNE2-derived tumors. Importantly, FM807 was able to induce EGFR, C-Raf and AKT degradation (Figure 4E and 4F). Western blot analysis of tumor tissues also showed that total EGFR, C-Raf, AKT and their phosphorylated forms were significantly decreased in all FM807-treated groups (Figure 4F). These data were consistent with the results obtained at the cellular level and confirmed the antitumor effect of FM807 in targeting EGFR and the downstream Raf/MEK/ERK and PI3K/AKT signaling pathways.

### FM807 physically binds to the N-terminal of Hsp90 and blocks Hsp90 ATPase activity *in vitro*

**Figure 5:**
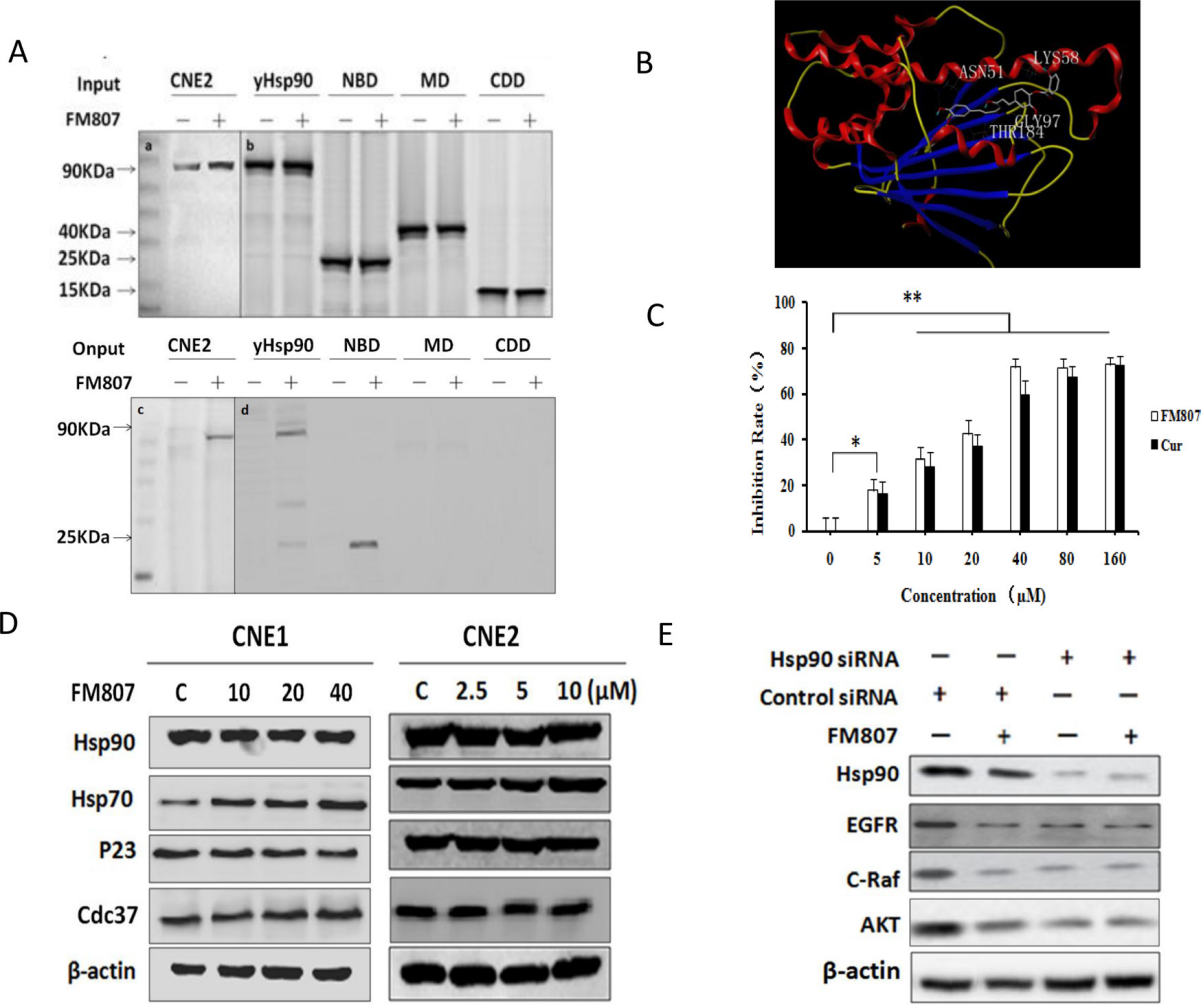
FM807 binds to Hsp90 and blocks Hsp90/client complexes (**A**) Western blot confirmed FM807 binds to the NBD of Hsp90. The “–” symbol of FM807-resin represents no drug-loaded affinity column, and the “+” symbol represents the drug-loaded affinity column. The test proteins were CNE2 cell lysate, His-tagged yeast Hsp90, NBD, MD and CDD of yeast Hsp90. Panels a and c show Human Hsp90 antibody; panels b and d show His-probe antibody. (**B**) Docking model of FM807 binding to N-terminal Hsp90. (**C**) The influence of FM807 on the ATPase activity of Hsp90 were determined by the malachite green reagent. The assay used 0.5 μM Hsp90 protein, 1 mM ATP, and FM807 or Cur at 0, 5, 10, 20, 40, 80 and 160 μM, to test the absorbance at 635 nm. Results are presented as means ± SD of three independent experiments. *P < 0.05: significant difference from control by ANOVA; **P < 0.01, very significant difference from control by ANOVA. (**D**) CNE1 and CNE2 cells were treated with FM807 for 24 h; Hsp90, Hsp70, Cdc37 and p23 protein levels were analyzed using western blotting. (**E**) CNE2 cells were treated by Hsp90 small interfering RNA (siRNA) and control siRNA for 6 h and incubated in normal growth medium for another 48 h. Whole-cell lysates were analyzed using western blotting with antibodies against EGFR, C-Raf, AKT and β-actin.

Previous work done by our lab has shown that Cur degrades Hsp90 client proteins, thereby suggesting that it is an Hsp90 inhibitor [12]. Moreover, EGFR, C-Raf and AKT are all key client proteins of Hsp90 and highly susceptible to Hsp90 inhibition [7]. Thus, we next examined the effect of FM807 on Hsp90. First, we created the FM807-loaded resins [22], and incubated with CNE2 cell lysate. To this, the lysate and resin were incubated with one of the following: (1) histidine-tagged full-length, (2) N-terminal nucleotide binding domain (NBD) (25 kDa), (3) the middle domain (MD) (40 kDa) or (4) C-terminal dimerization domain (CDD) (15 kDa) of yeast Hsp90. This affinity-based screen showed that FM807 bound to NBD of Hsp90, but not to MD or CDD (Figure 5A).

We then used Molsoft ICM 3.5a to model the interaction between Hsp90 (Protein Data Bank ID 2CCS) and FM807. As shown in Figure 5B, the side chain of ASN51, LYS58, GLy97 and THR184 could potentially form four hydrogen bonding interactions with the NBD of Hsp90 (Figure 5B).

Since the chaperone function of Hsp90 is dependent on ATP binding, and ASN51 and GLY97 are both involved in the ATP binding sites of Hsp90 [32], we next tested the influence of FM807 on the ATPase activity of Hsp90. Our colorimetric assay for inorganic phosphates revealed that FM807 could inhibit the ATPase activity of Hsp90 in a concentration-dependent manner. Importantly, the inhibition of FM807 on Hsp90 ATPase was greater than that of Cur (Figure 5C).

Hsp90 requires a series of co-chaperones such as cell division cycle protein 37 (Cdc37), Hsp70 and p23 to form a super-chaperone complex. This complex is then released at various time points to regulate the folding, assembly and maturation of Hsp90 client proteins [33]. Given this, we sought to further investigate the influence of FM807 on the interactions between Hsp90 and its co-chaperones using CNE1 and CNE2 cells. Hsp90, Cdc37 and p23 levels all showed no distinct changes as a function of increasing FM807 concentration. However, Hsp70 is a mark of Hsp90 inhibition, and was increased by treatment after FM807 treatment (Figure 5D). Furthermore, after depletion of Hsp90 protein by siRNA, FM807 was unable to induce EGFR, C-Raf and AKT protein degradation, suggest that Hsp90 protein is a direct target of FM807 (Figure 5E).

### FM807 suppresses nuclear β-catenin signaling pathway

**Figure 6:**
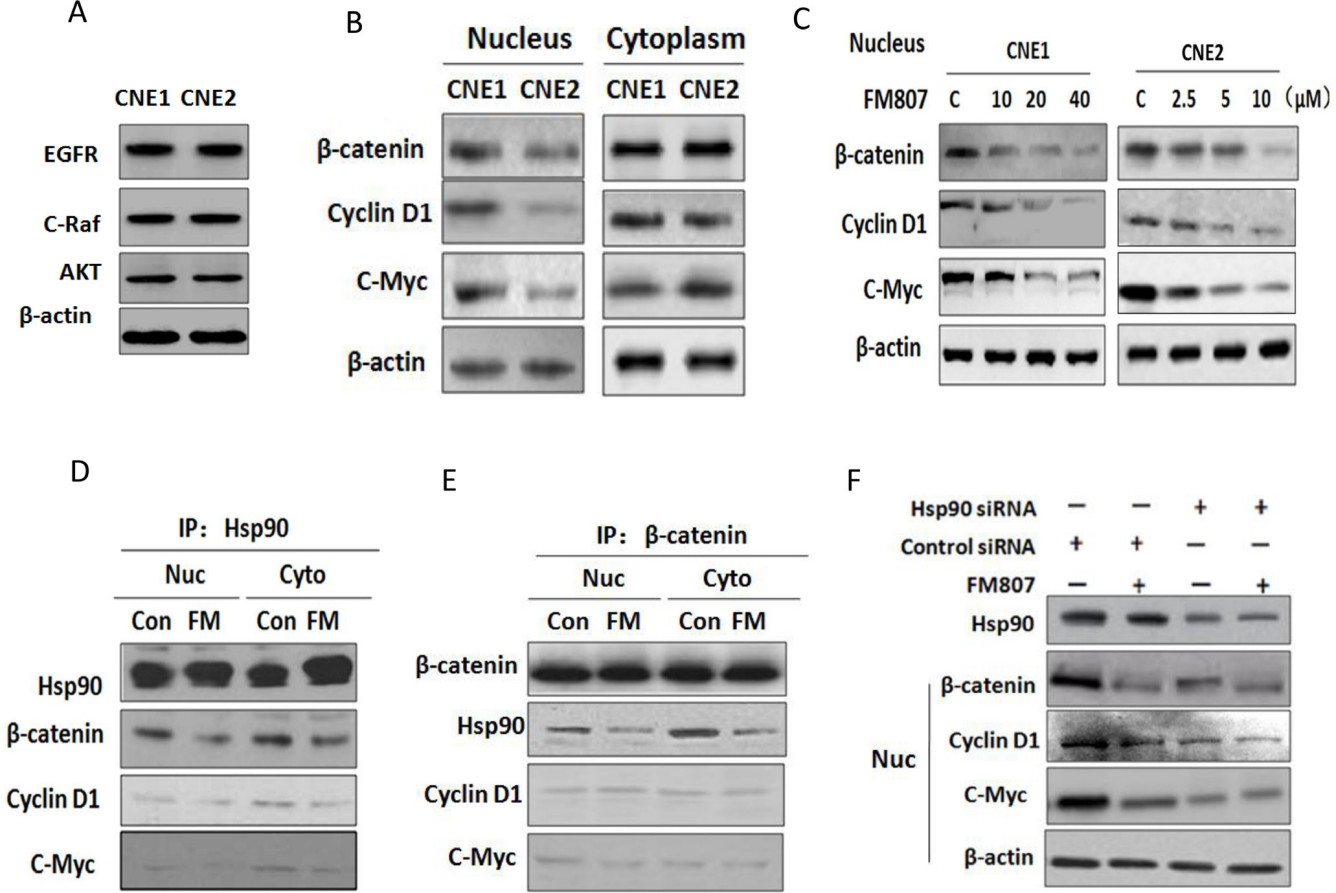
FM807 suppresses nuclear β-catenin signaling pathway (**A**) Expression of EGFR, C-Raf, and AKT in CNE1 and CNE2 using western blot. β-actin was used as a loading control. (**B**) Cytoplasmic and nuclear expression of β-cantenin, Cyclin D1 and c-Myc in CNE1 and CNE2 cells. (**C**) CNE1 and CNE2 cells were treated with increasing FM807 concentrations for 24 h. β-cantenin, Cyclin D1 and c-Myc levels were analyzed using western blot. (**D**) and (**E**) CNE2 cells were treated with 10 μM FM807 for 6 h. Hsp90 and β-cantenin were immunoprecipitated from whole-cell lysates (500 μg each) with anti-Hsp90 or anti-β-cantenin antibodies. (**F**) CNE2 cells were knocked down by Hsp90 siRNA and control siRNA with the transfection complexes for 6 h and incubated in normal growth medium for another 48 h. Nuclear fractions were analyzed using western blot with antibodies against β-cantenin, Cyclin D1, c-Myc and β-actin.

The data showing a greater sensitivity of CNE2 to FM807 than CNE1 were both interesting and unexpected. We next wonder: Is there differential Hsp90 clients expression that underlies the difference in FM807 chemosensitivity between CNE1 and CNE2 cell lines? To answer this question, we used western blot analysis to determine protein levels of EGFR, C-Raf and AKT of CNE1 and CNE2 cells. Counter to our hypothesis, the result showed that there were no significant differences of EGFR, C-Raf and AKT protein levels between CNE1 and CNE2 (Figure 6A). This finding indicating that EGFR and its downstream signaling might not be the reason for the observed differences in chemosensitivity.

It has been reported that CNE1 is more radioresistant than CNE2, and that the transcriptional activity of β-catenin is closely associated with radiation resistance in cancer progenitor and stem cells [34, 35]. β-catenin is the key mediator of canonical signaling in the Wnt/β-catenin pathway, and a hallmark of Wnt/β-catenin signaling activation is the stabilization and nuclear translocation of cytoplasmic β-catenin [36]. Intranuclear β-catenin then interacts with numerous downstream proliferation signals, including c-Myc and Cyclin D1 [37].

Western blot analysis was used to determine cytoplasmic and nuclear protein levels in both CNE1 and CNE2 (Figure 6B). Although there were no significant differences in cytoplasmic levels of β-catenin, Cyclin D1 or c-Myc between CNE1 and CNE2 cell lines, results showed significantly higher nuclear expression of β-catenin, Cyclin D1 or c-Myc in CNE1 cells relative to CNE2 cells. We next determined the effect of FM807 on the nuclear transcription factors in CNE1 and CNE2 cells. Additional western blot analysis further confirmed decreases of nuclear β-catenin, CyclinD1 and c-Myc in both CNE1 and CNE2 cells following FM807 treatment (Figure 6C). Therefore, the differences in FM807 sensitivity between CNE1 and CNE2 cell lines might be associated with intranuclear protein expression. However, the exact relationship between FM807 chemosensitivity and nuclear transcription factor levels still remains unclear.

It has been reported that Hsp90 is an essential molecular chaperone for nuclear transport, and Hsp90 could modulate both β-catenin and its phosphorylation levels [38]. Given this, β-catenin might be a client of Hsp90 [39], therefore meaning that FM807-induced depletion of these nuclear transcription factors would likely operate through Hsp90 inhibition. To confirm this, co-immunoprecipiation experiments were performed with an anti-Hsp90 antibody in both cytoplasmic and nuclear fractions isolated from CNE2 cells (Figure 6D). Results showed that nuclear β-catenin was successfully detected in the Hsp90 co-immunoprecipitation, indicating that β-catenin might be chaperoned by Hsp90. Reverse immunoprecipiation of β-catenin confirmed coimunoprecipiation of Hsp90 (Figure 6E). Nuclear Cyclin D1 and c-Myc bands were also visible in both the Hsp90 and β-catenin immunoprecipitation, demonstrating the interaction between β-catenin, CyclinD1 and c-Myc (Figure 6D and 6E). To further clarify the interaction between Hsp90 and nuclear β-catenin, we downregulated Hsp90 expression in CNE2 using Hsp90 siRNA transfection (Figure 6F). Results showed that FM807 was unable to deplete nuclear β-catenin, CyclinD1 or c-Myc protein levels after Hsp90 knockdown, suggesting that FM807 likely reduces the nuclear translocation of these proteins by inhibiting Hsp90 chaperone functioning.

## DISCUSSION

Hsp90 is a highly conserved and abundant molecular chaperone found in all eukaryotes [7]. Hsp90 is required for proper folding and maturation of most oncogenic proteins which can then aberrantly activate multiple signaling pathways [40–42]. Therefore, the molecular chaperone Hsp90 is an attractive target for cancer therapy.

In the present study, we have shown that the Curcumin analogue FM807, has better anti-proliferative abilities than Curcumin (Figure 1B and 1C), has both *in vitro* and *in vivo* antitumor effects in NPC cells, and displays selective inhibition of proliferation in non-malignant cells and two NPC cell lines. We have also shown that FM807 is an Hsp90 inhibitor that directly binds to the N-terminus of Hsp90 (Figure 5A and 5B) and interrupts Hsp90/client complexes (Figure 5E). Inhibition of cell growth and induction of necrotic/late apoptotic cell death and G1 phase arrest (Figure 2) by FM807 was also shown in both well-differentiated CNE1 and poorly-differentiated CNE2 cells.

Overexpression of EGFR, one of the most sensitive Hsp90 client proteins, has been reported in 85% of NPC tissues and is associated with poorer patient prognosis [43–45]. EGFR is the tyrosine kinase receptor of the natural ligand EGF, with EGFR activating numerous signaling cascades associated with proliferation, migration, and drug resistance, notably the Raf/MEK/ERK and PI3K/AKT pathway [46]. Both *in vitro* and *in vivo* analyses revealed that FM807 potently inhibited EGFR and its downstream signaling (Figures 3A, 3B, 4C and 4D). Moreover, FM807 was found to suppress EGF-stimulated EGFR/Raf/MEK/ERK and PI3K/AKT cascades and to simultaneously downregulate total EGFR, C-Raf, and AKT levels. This observation raises the possibility that FM807 may have clinical benefit for NPC patients with high EGFR expression.

Moreover, FM807 showed better inhibition in poorly-differentiated CNE2 cells compared to well-differentiated CNE1 cells. It also had better antitumor effects in CNE2 xenografts models when compared to CNE1-derived models (Figure 4A and 4C), with IC_50_ values of 3.59 and 25.75 μM, respectively. This is a promising finding, as CNE2 cells are more malignant than CNE1 [1].

To better clarify how FM807 might lead to such different chemosensitivities, we used western blot analysis to determine cytoplasmic and nuclear protein levels. Our results revealed higher nuclear β-catenin, Cyclin D1 and c-Myc levels in CNE1. β-catenin is a key effector of Wnt signaling, and nuclear β-catenin levels are increased in 92% of NPC tumors, thus rendering it an important component of NPC development [27]. Aberrant intranuclear β-catenin activates numerous downstream transcription factors, including Cyclin D1 and c-Myc, to promote cell proliferation and differentiation. Cyclin D1 is response for cell progression through G1 phase, and overexpression of Cyclin D1 enables cells with unrepaired structural or genomic damage to traverse the G1/S checkpoint, thus increasing the risk of tumor formation [47–49]. C-Myc is also critical to the regulation of G1/S phase proteins, and upregulation of c-Myc is a common occurrence in cancers for chromosomal translocations and point mutations [50, 51]. Decreases in nuclear β-catenin, Cyclin D1 and c-Myc were observed in both CNE1 and CNE2 cells (Figure 6B, 6C and 6D), These decreases likely contributed to a decrease in the DNA repair and replication capacity resulting from FM807 inhibition of Hsp90, thus preventing cell from transitioning into the S phase and arresting it in the G1 phase (Figure 2).

## MATERIALS AND METHODS

### Cell lines and reagents

Well-differentiated and poorly-differentiated NPC cell lines CNE1 and CNE2 (obtained from Fujian Provincial Tumor Hospital of China) were cultured in RPMI 1640 supplemented with 10% heat-inactivated fetal bovine serum (FBS) (GIBCO, Gaithersburg, MD, USA), 100 IU/ml penicillin and 100 IU/ml streptomycin. Human epithelial cell line Rhek (obtained from the Medical Center of Minnesota University) was grown in MEM supplemented with 10% fetal bovine serum, 100 IU/ml penicillin, 100 IU/ml streptomycin and 1% L-glutamine. FM807 (purity ≥ 98.5%) was synthesized from Curcumin (obtained from the Third Reagent Factory of Shanghai, China) at the New Drug Institute of Fujian Medical University, China. All other chemical reagents were obtained from Sigma Aldrich.

Primary antibodies against β-actin, cleaved caspase-3, caspase-3, Hsp90, Hsp70, p23, Cdc37, CDK4, CDK2, Cyclin D1, Cyclin A, Cyclin E, Bax, Bcl-2, Bcl-xl, EGFR, C-Raf, AKT, β-catenin, c-Myc, phospho-EGFR (P-EGFR), phospho-C-Raf (P-C-Raf), phospho-AKT (P-AKT) were obtained from Cell Signaling Technology. MTS was obtained from Promega. The Annexin V: fluorescein isothiocyanate (FITC) Apoptosis Detection Kit I was purchased from BD Biosciences.

### Preparation of Hsp90 proteins

Full-length (1-732, 90 kDa), N-terminal nucleotide binding domain (NBD) (1-236, 25 kDa), the middle domain (MD) (272-617, 40 kDa) and C-terminal dimerization domain (CDD) (629-732, 15 kDa) versions of recombinant yeast Hsp90 proteins with histidine-tag were prepared as previously described [22].

### Chemoproteomics assay

CNBr-activated Sepharose^™^4B (GE Healthcare) was swelled in 1mM HCl and washed with coupling buffer (0.1 M NaHCO_3_, 0.5M NaCl, pH = 8.3). After the resin was swelled, washed, and added to the coupling buffer, FM807 was dissolved in dimethyl sulfoxide (DMSO) and added to the resin (up to 10 μmoles per mL of medium). The mixture was rotated end over end for 4 h at room temperature, and excess ligand was removed by washing with coupling buffer. Any remaining active groups were blocked for 2 h at room temperature with the capping solution. The column was then equilibrated with coupling buffer for 1 h. Hsp90 test proteins were added to the resin, the mixture was rotated overnight at 4°C. Any unbound proteins were removed by washing buffer. Loading buffer was added to the resin, boiled for 10 min, and separated using 10% sodium dodecyl sulfate polyacrylamide gel electrophoresis. Protein separation was followed by western blotting for the protein of interest.

### MTS assay

Cells were seeded in 96-well plates (5 × 10^3^/well), and treated with various concentrations of Cur and FM807. Cell viability was assessed after treatment using an MTS assay (Promega) according to the manufacturer's instructions. Results were calculated based on the principle that the number of living cells was proportional to MTS absorbance at 490 nm. All results are presented as means ± SD from three independent experiments. Inhibition graphs used mean values obtained from each concentration relative to control values. Half maximal inhibitory concentrations (IC_50_) were calculated using PASWstatistics 18 (SPSS, Inc).

### Colony formation assay

Cells were plated in 24-well plates (500/well) and treated with various concentrations of Cur and FM807. After two weeks of treatment, colonies were staining with Giemsa for 10 min, and counted using light microscopy. Colonies were defined as ≥ 50 cells.

### Cell cycle analysis

Cells were seeded in 6-well plates and treated with various concentrations of FM807 for 24 h. Cells were harvested, washed with phosphate-buffered saline (PBS), and fixed in 70% ethanol overnight at −20°C. After additional washing, cells were incubated with RNase A (20 μg/mL) at 37°C for 30 min, stained with propidium iodide (100 μg/mL; Sigma Aldrich) for 10 min, and analyzed with flow cytometry (BD FACSC autoTM II).

### Apoptosis assay

Apoptosis was determined using the Annexin-V:FITC Apoptosis Detection Kit I (BD Biosciences) according to the manufacturer's protocol. Briefly, DMSO or FM807 -treated cells were collected via centrifugation and washed once with PBS. Cells were stained with fluorescein and propidium iodide for 15 min at room temperature and subsequently analyzed by flow cytometry.

### Immunoprecipitation

Samples (500 μg of total protein) were incubated overnight with 2 μg of primary antibody at 4°C, after which 20 μL of protein A Mag Sepharose^™^ (GE Healthcare, UK) were added to the mixture. The mixture was then rotated for 2 h at 4°C. The immunoprecipitated protein complexes were then washed once with lysis buffer and twice with ice-cold PBS. The resulting supernatant was discarded and the antibody/protein complexes were resuspended in 30 μL of loading buffer and boiled for 5 min. Proteins were then separated with 10% sodium dodecyl sulfate polyacrylamide gel electrophoresis and assayed with immunoblotting.

### Colorimetric determination of ATPase activity

Malachite green reagent [50, 51] was prepared on the day of use, which contained malachite green (0.0812%, w/v), polyvinyl alcohol (2.32%, w/v, dissolves with difficulty and requires heating), ammonium molybdate (5.72%, w/v, in 6 M HCl), and argon water mixed in a ratio of 2:1:1:2 to a golden yellow solution. The assay buffer consisted of 100 mM Tris-HCl, 20 mM KCl, and 6 mM MgCl_2_, with a pH of 7.4. The experiments were performed in 100 μL of test solution containing 80 μL of malachite green reagent. The test solutions contained 0.5 μM Hsp90 protein, 1 mM ATP, and different concentrations of FM807 or vehicle (DMSO) were shaken for 15 min at room temperature. Absorbance values were derived from the plate reading at 635 nm.

### Small interfering RNA (siRNA) gene knockdown

Cells were seeded in antibiotic-free normal growth medium supplemented with FBS. Single siRNA oligonucleotides (obtained from Santa Cruz Biotechnology) targeting human Hsp90α/β (sc-35608) and control siRNA (sc-37007) were diluted in siRNA Transfection Medium (sc-36868) and mixed with siRNA Transfection Reagent (sc-29528) according to the manufacturer's protocol. Cells were first incubated with the transfection complexes for 6 h followed by normal growth medium for 48 h. Cells were then treated with either DMSO or FM807 for 24 h before cell lysates were prepared and analyzed using western blot.

### Quantitative real-time PCR

Total RNA extraction was performed with TRIzol reagent (Life Technologies Corporation), and first strand cDNA was synthesized using 1 μg of total RNA treated with avian myeloblastosis virus (AMV) reverse transcriptase (Promega) according to the manufacturer's instructions. Quantitative real-time reverse transcription polymerase chain reaction (RT-PCR) analysis was performed in triplicate with FastStart Essential DNA Green Master (Roche) using LightCycler 96 (Roche). The ΔΔCT method was used to calculate relative expression. Primer sequences used in RT-PCR were as follows: human EGFR (forward 5′-AAGGCTGTCCAACGAATGGG -3′ and reverse5′-CCTCTCCTGCAGCAGCCTC-3′), AKT (forward 5′-TTGAGAGAAGCCACGCTGT-3′ and reverse 5′-CGGAGAACAAACTGGATGAA-3′), C-Raf (forward 5′-CACCTCCAGTCCCTCATCTG-3′ and reverse 5′-CTCAATCATCCTGCTGCTCA-3′), and GAPDH as control (forward 5′-AGAAGGCTGGGGCTCATTTG-3′ and reverse 5′-AGGGGCCATCCACAGTCTTC-3′).

### Preparation of cell lysates and cell fractions

For whole cell lysates, 1 × 10^7^ cultured cells were harvested and washed twice with ice-cold PBS, and then lysed for 15 min at 4°C with 500 μL lysis buffer (10 mM Tris-HCl pH 8.0, 1mM EDTA, 2% sodium dodecyl sulfate (SDS); 5mM dithiothreitol (DTT); 10mM phenylmethanesulfonyl fluoride (PMSF, Sigma Aldrich), a cocktail of protease and phosphatase inhibitors (Roche, Indianapolis, IN), and PhosSTOP (Roche Diagnostics), then centrifuged at 12,000 rpm for 10 min and the supernatant was collected and stored at −70°C until late use.

For the preparation of cytoplasmic and nuclear factions, 1 × 10^7^ cultured cells were washed with PBS and suspended in 200 μL of lysis buffer (10 mM Hepes, pH7.9; 10 mM KCl; 0.1 mM EDTA; 0.1 mM EGTA; 1mM DTT; 0.5 mM PMSFand protease inhibitor cocktail). Cells were incubated on ice for 15 min, after which 6.5 μL of 12.5% NP-40 were added; the contents were mixed and then centrifuged for 1 min at 12,000 rpm. The supernatant was saved as the cytoplasmic fraction. The pellet was resuspended in 12.5 μL of ice-cold nuclear extraction buffer (20 mM Hepes, pH7.9; 0.4 M NaCl; 1 mM EDTA; 1 mM EGTA; 1mM DTT; 1 mM PMSF and protease inhibitor cocktail) and incubated on ice for 40 min with mixing every 10 min. The solution was then centrifuged at 12,000 rpm for 5 min at 4°C. The resulting supernatant was saved as the nuclear fraction. The cytosolic and nuclear fractions were stored at −70°C until later use.

### Western blot analysis

Protein concentration was determined using the BCA Protein Assay Kit (Thermo Scientific) according to the manufacturer's instructions. Equal amounts of protein were separated using SDS-PAGE, transferred to PVDF membranes and blotted with specific primary antibodies. Proteins were detected via incubation with horseradish peroxidase-conjugated secondary antibodies and visualized with SuperSignal WestPico (Thermo Scientific). All Western blots were repeated at least three times to ensure replicability.

### Tumor xenografts

BALB/c (nu/nu) athymic male mice, aged 4–5 weeks and weighing approximately 18–20g, were purchased from Shanghai Institutes for Biological Sciences, Chinese Academy of Sciences. For the xenografts, 6 mm^3^ tumor fragments were implanted into the subcutaneous tissue of the axillary region of mice using a trocar needle after which mice were randomly divided into control and treatment groups (*n* = 6). Animals were given daily intragastric (i.g.) administration of either vehicle or FM807 (incorporated drug solid dispersions with poloxamer188 and further diluted in physiological saline) daily. Tumor volumes were calculated using the following ellipsoid formula: [D × (d^2^)]/2, in which D is the large diameter of the tumor, and d is the small diameter. Tumor volumes were plotted as means ± SD. All animal experiments were approved by the Animal Care and Use Committee, Fujian Medical University, China.

### Immunohistochemistry (IHC)

Briefly, paraffin sections were processed according to the following steps: dewaxing, endogenous peroxidase blockade using H_2_O_2_ treatment, serum blockade before treatment with primary antibody (1:100) at 4°C and biotin-conjugated secondary antibody (1:100) at 37°C. Visualization was performed using DAB, followed by dehydration, transparentization and tissue mounting. The number of positive targets was counted using 400 × microscope, and analyzed by HMIAS high-definition color medical image analysis system (Wuhan, China).

### Statistical analysis

ANOVA was employed for comparisons across multiple groups, and data were reported as mean ± SD. Statistical analysis was performed using PASWstatistics 18 (SPSS, Inc). *P* < 0.05 was considered to be statistically significant.

## CONCLUSIONS

As a novel analogue of curcumin, FM807 inhibits NPC cell growth, and induces G1 phase arrest and necrotic/late apoptotic cell death in CNE1 and CNE2 cells *in vitro*. Binding of FM807 to the N-terminus of Hsp90 likely blocks formation of Hsp90/client complexes, resulting in degradation of the Hsp90 client protein EGFR and inhibition of the downstream Raf/MEK/ERK and PI3K/AKT pathway. Different intranuclear transcription factors such as β-catenin, Cyclin D1 and c-Myc, would likely cause disparate FM807 chemosensitivities. However, FM807 depletes these intranuclear transcription factor levels by inhibiting Hsp90 nuclear chaperone functioning. Based on these findings of tumor suppressive effects and low toxicity in xenograft mice, this study provides a preclinical basis for the further development of FM807 as a novel therapeutic agent in the treatment of NPC.
